# Ureteral access sheaths: a comprehensive comparison of physical and mechanical properties

**DOI:** 10.1590/S1677-5538.IBJU.2017.0575

**Published:** 2018

**Authors:** Nishant Patel, Manoj Monga

**Affiliations:** 1 Department of Urology, Glickman Urological and Kidney Institute, Cleveland Clinic Foundation, USA

**Keywords:** Ureteroscopy, Ureter, instrumentation [Subheading]

## Abstract

**Introduction:**

Ureteral access sheaths (UAS) facilitate flexible ureteroscopy in the treat- ment of urolithiasis. The physical properties of UAS vary by manufacturer and model. We compared three new UAS: Glideway (GW, Terumo, 11/13Fr, 12/14Fr), Pathway (PW, Terumo 12/14F) and Navigator HD (NHD, Boston Scientific, 11/13Fr, 12/14Fr) in the domains of safety characteristics, positioning characteristics, lubricity and radio- opacity.

**Materials and Methods:**

*In vitro* testing of the three UAS included safety testing-tip perforation force, sheath edge deformation and dilator extraction forces. Positioning characteristics tested included tip bending, stiffness (resistance to coaxial buckling forces), kinking (resistance to perpendicular forces), and insertion forces. Lubricity was assessed by measured frictional forces of the outer sheath. Finally, radio-opacity was tested utilizing fluoroscopic imaging of the three 12F sheaths and inner dilators.

**Results:**

The PW (0.245 lb) and GW (0.286 lb) required less force for tip perforation compared to the NHD (0.628 lb). The NHD sheath edge deformation was mild compared to more severe deformation for the PW and GW. The PW (1.008 lb) required greater force than the GW (0.136 lb) and NHD (0.043 lb) for inner dilator removal. The GW (3.69 lbs) and NHD (4.17 lb) had similar inner dilator tip stiffness when bent, while the PW had the weakest inner dilator tip, 1.91 lbs. The PW (0.271 lb) was most susceptible to buckling and kinking (1.626 lb). The most lubricious UAS was the NHD (0.055 lbs for 12F). The NHD (0.277 lbs) required the least insertional force through a biological model and possessed the greatest radio-opacity.

**Conclusions:**

Comparison of different commercially available UAS in various sizes reveals that there are mechanical differences in sheaths that may play a role clinically. The Terumo sheaths' (GW and PW) were outperformed by the Boston Scientific NHD in simulating safety, ease of use and radio-opacity.

## INTRODUCTION AND OBJECTIVES

**Figure 1:**
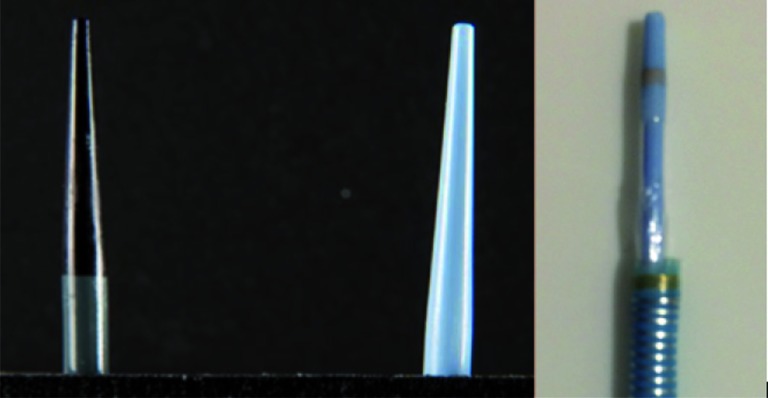
(From left to right) Boston Scientific Navigator HD, Terumo Glideway, and Terumo Pathway.

Ureteral access sheaths (UAS) are commonly used to facilitate flexible ureteroscopy in the treatment of urolithiasis. Reports have highlighted the practical role of the UAS in reducing operative times, allowing multiple passes of instruments, and protecting the ureteroscope (1–4). Further, the UAS has the capacity to improve visualization and reduce intra-renal pressure during ureteroscopy (5, 6). A few studies have observed that UAS can improve stone free rates, though the level of evidence is low (7, 8). Selection of the UAS among the choices of manufacturers and models typically depends on physician familiarity, cost, and size of ureteroscope (9). The physical properties of ureteral access sheaths vary by manufacturer and model, and these specific characteristics may play a role in their clinical applicability. Dilator tip shape, flexibility, and ease of extraction may affect ureteral safety during sheath advancement and extraction (10). Sheath strength and ability to withstand a diversity of directional forces may impact utility during UAS insertion. In an *in vitro* study, we examined the physical and mechanical properties of the UAS that may impact ergonomics, efficacy, and patient safety. Specifically, we compared three new UAS (Figure-1); the Glideway (GW, Terumo, 11/13F, 12/14F), the Pathway, a distinct balloon-expandable sheath (PW, Terumo, 12/14f), and Navigator HD (NHD, Boston Scientific, 11/13F, 12/14F).

## MATERIALS AND METHODS

**Figure 2:**
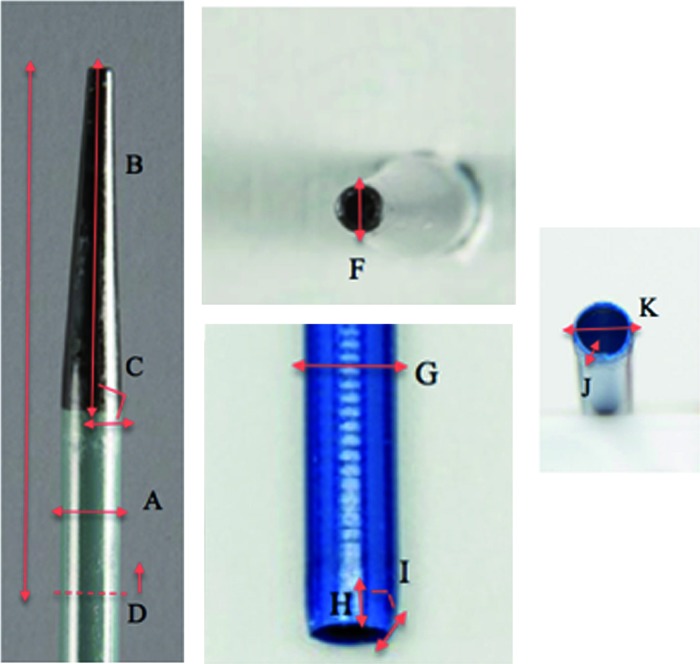
Tip contour and sheath characteristics. A) Diameter of inner dilator; B) Taper length of inner dilator; C) Taper grade of inner dilator; D) portion of inner dilator exposed from sheath; E) Tip Length; F) Leading edge of inner dilator; G) Diameter of outer sheath; H) Taper length of outer sheath; I) Taper grade of outer sheath; J) Thickness of outer sheath; K) Leading edge outer sheath.

*In vitro* testing of the Glideway (Terumo, NJ, USA, 11/13F, 12/14F), the Pathway, a balloon expandable UAS (Terumo, NJ, USA, 12/14f), and Navigator HD (Boston Scientific, MA, USA, 11/13F, 12/14F) was conducted to compare physical and mechanical characteristics. We have previously described our methodological techniques for single-wire ureteral access sheaths (10). The GW and NHD were selected as examples of new models of the traditional UAS, with a tapered inner dilator and hydrophilic outer sheath, while the PW represents a unique balloon-expandable sheath design, meant to minimize the sheath diameter. Safety characteristics (tip perforation forces, sheath edge deformation, dilator extraction forces), positioning characteristics [tip bending, stiffness (resistance to coaxial buckling forces), kinking (resistance to perpendicular forces), insertion forces], and lubricity (frictional forces of outer sheath) were examined. Further testing was performed on the PW utilizing an *ex vivo* model (pig ureters) to assess risk for mucosal avulsion for a balloon-expandable device during extraction of the sheath. Tip bending, sheath buckling, dilator removal and frictional forces were measured with an Amplatz Superstiff guidewire (Boston Scientific, MA, USA) inserted through the inner lumen of the inner dilator of the sheath. Inner dilator tip contours and sheaths physical characteristics (Figure-2) were measured using digital calipers (Niko 01407A, China). To assess radio-opacity, fluoroscopic images were taken of the three 12F sheaths and inner dilators. This radio-opacity test was per-formed utilizing a C-arm (Siemens Arcadis mobile C-arm, Germany) at a distance of 38cm from the intensifier to the sheath with a voltage of 99kV and a tube current of 5.4 mA.

### Safety testing

#### Tip perforation

**Figure 3:**
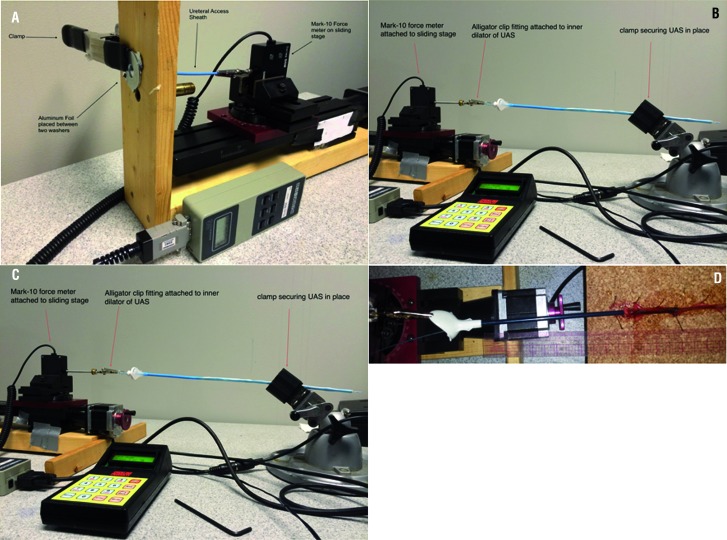
A) Tip perforation; B) Edge Deformation; C) Dilator Removal; D) Extraction Force for Pathway (*Ex* vivo model).

The inner dilator was mounted on a motorized sliding stage. The dilator end was attached at a length of 10 cm via an alligator clip. The alligator clip was fitted to a digital force meter, which continuously measured force (Mark-10 Corp, NY, USA). The stage was advanced until a foil membrane (0.016 mm thickness, standard) was punctured. The maximum force at perforation of the foil membrane was recorded in pounds (Figure-3A). A total of 5 trials were run for each sheath and averaged.

#### Edge Deformation

The force meter was attached to the sheath at a length of 10 cm. The working end of the sheath was placed directly against a BegoStone®, a synthetic, commercially-available, hard stone phantom made from dental plaster (BEGO USA, Smithfield, RI). The sheath was advanced until a set force was achieved (2.5 lbs). Photos were taken to qualitatively examine outer sheath edge deformation (Figure-3B).

#### Dilator Removal

The outer sheath was secured at tip end via an adjustable vice and the inner dilator (locking mechanism disengaged) was fixed to a motorized, continuous, digital force meter (Mark-10). The stage was retracted for 7s at 5 mm/s, and average force for a 5s interval was calculated (1-6s) as the dilator was removed (Figure-3C). A total of 5 trials were run for each sheath and averaged.

#### Extraction Force for Pathway Sheath (Ex Vivo Model)

Superstiff Guidewires were inserted through *ex vivo* ureters (pinned to cork board) and each access sheath was wetted and placed over the guidewire and advanced until in contact with 15 cm of the lumen of the ureter. Devices were deployed by inflation of the inner balloon, balloon withdrawn and outer sheath left in place for 30 minutes (3 trials each), then attached to a load cell on a stepper motor and extracted at a constant rate, recording the maximum force. Same methods were used while devices were left in place for approximately 60 minutes, for 5 trials each (Figure-3D).

### Positioning Testing

#### Tip Bending

**Figure 4:**
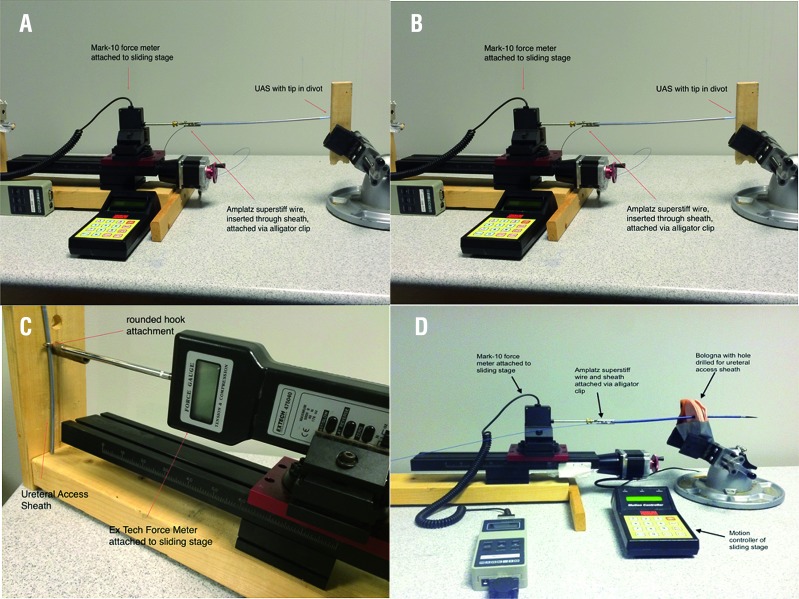
A) Tip Bending; B) Sheath Buckling; C) Kinking; D) Insertion force experiment (sheath advanced until midway across UAS).

The dilator tip was cut to the length that is typically exposed from the outer sheath. The tip was mounted to a stage and secured to the digital force meter with the alligator clip attachment. The tip was secured in a 2 mm divot in a wooden block and the stage was advanced 1cm recording maximum force (Figure-4A).

#### Sheath Buckling

The ureteral access sheath was mounted to a stage and attached to the Mark-10 force meter via an alligator clip at a length of 25 cm from the tip. The tip of the sheath was placed in a 2 mm divot in a wooden block and the stage was advanced 5 cm recording maximum force as the sheath buckled (Figure-4B). A total of 5 trials were run for each sheath and averaged.

#### Kinking

The ureteral access sheath was secured vertically against a wooden block. A digital force meter (EX Tech Instruments, 475040, Taiwan), equipped with rounded end attachment was placed perpendicular, flush with the sheath. The rounded end was advanced in 1 mm increments up to 4mm. Maximum force for each increment was recorded as the sheath began to kink (Figure-4C).

#### Insertion Force with Biological Model

The Amplatz superstiff guide wire was place through the device, and the wire was inserted through the biological model (Oscar Meyer Bologna, 45 mm). The sheath was mounted to a motorized sliding stage and attached to a digital force meter via an alligator clip fitting. The sheath tip was placed flush with the biological model, and the device was advanced at a constant speed penetrating through the biological tissue. The GW and NHD were advanced from tip to mid-shaft, recording maximum force. The PW was advanced in several sections to determine where the greatest force may occur, including 1) until just beyond the tip of the device, 2) from the tip of the device to the middle deflated section, and 3) from the deflated section to the rigid end (Figure-4D).

#### Frictional Forces with Biological Model

Biological tissue (Oscar Meyer Bologna, 45 mm) was secured via an adjustable vice. Sheaths were soaked in water and inserted perpendicularly through the biological model up to 3 cm from the proximal end for the start of the experiment. The sheath was then retracted for 7s at 5 mm/s while attached to motorized, continuous, digital force meter (Mark-10). Average force was calculated for 5 seconds of the test (1-6s). The PW sheath was extracted as designed; that is, the balloon was inflated after insertion through the biological model, the balloon was then removed, and the extraction force of the remaining outer sheath was measured (Figure-4D).

## STATISTICAL ANALYSIS

Descriptive statistics were calculated for all sheaths in each experiment; average forces and standard deviation are displayed in Table-1. UAS were divided into groups based on inner diameter (12F and 11F). Student *t* tests were performed to compare two individual sheaths of the same size (12/14F, 11/13F) for all experiments, and ANOVA was used to compare 3 or more sheaths. Statistical significance was determined at P<0.05.

**Table 1:** Outer Sheath Characteristics.

Outer Sheath	Diameter (mm)	Taper length (mm)	Taper grade	Taper grade (degrees)	Tip length (mm)	Leading edge (mm)
GW 12	4.68	n/a	n/a	n/a	0.25	4.24
PW 12	4.2	n/a	n/a	n/a	0.32	4.86
NHD 12	4.82	2.85	0.065	3.714	0.27	4.45
GW 11	4.33	n/a	n/a	n/a	0.25	3.85
NHD 11	4.43	2.12	0.066	3.778	0.3	4.15

**n/a** = not available

## RESULTS

### Tip Contour and Sheath Characteristics

Tip contour and sheath measurements are displayed in Tables 1 and 2. The GW inner dilator tip had the longest taper length and most gradual taper grade, making it the sharpest of the sheaths. The NHD inner dilator had the greatest taper grade, making it blunter than the GW and the PW. The diameter of the NHD's outer sheath was larger than both the PW and the GW. The PW's outer sheath was the thickest, likely due to the balloon-distension design, while the GW was the thinnest. The NHD outer sheath had a mild taper, while the Terumo GW and PW sheaths' did not.

**Table 2:** Tip Contour Measurements.

Inner Dilator	Diameter (mm)	Taper length (mm)	Taper grade	Taper grade (degrees)	Tip length (mm)	Leading edge (mm)
GW 12	4.02	26.72	0.041	2.38	28.66	1.8
PW 12	1.53	8.21	0.045	2.58	48.8	1.44
NHD 12	3.97	21.25	0.056	3.19	25.5	1.6
GW 11	3.72	24.96	0.040	2.32	26.83	1.7
NHD 11	3.61	19.6	0.049	2.83	24.78	1.7

### Radio-opacity (Figure 5)

**Figure 5:**
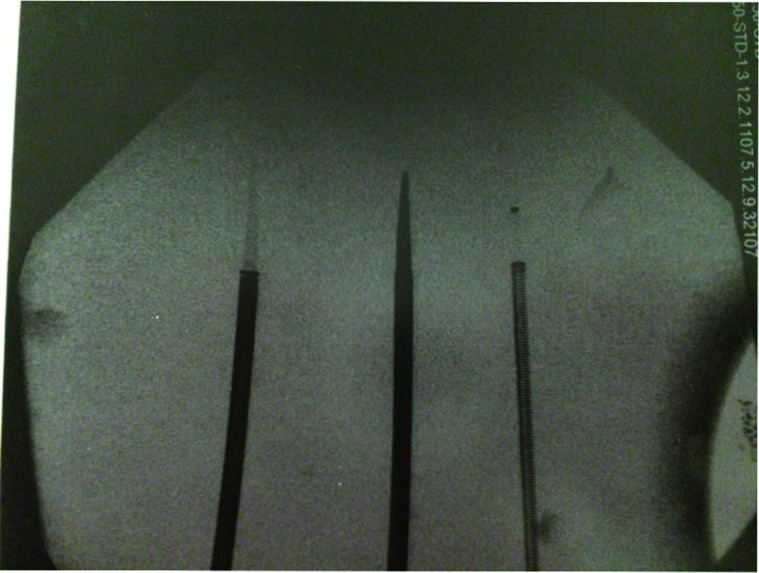
Radio-opacity. from left to right: GW, NHD, PW.

The NHD was uniformly radiopaque, and the entire inner dilator tip was clearly visible on X-ray. The GW had a radiopaque marker at the distal end of its outer sheath, but the leading edge actually extended beyond this marker. In addition, the GW's inner dilator was barely visible on X-ray. The PW also had a distal outer sheath marker, with a small portion of the sheath's leading edge extending beyond this marker. Unlike the GW, the PW's inner dilator also had a radiopaque marker at the distal tip, although the remainder of the inner dilator was difficult to visualize on X-ray.

### Safety Characteristics- Table-3

**Table 3:** Mean forces (lbs) for safety, positioning and lubricity testing.

	Experiment	GW 11	NHD 11	t Test p value	GW 12	NHD 12	PW 12	ANOVA Test p value for 12F
**Safety**	**Tip Perforation**	0.398±0.252	0.67±0.103	0.008*	0.286±0.094	0.628±0.204	0.245±0.067	9.73X10^-7^ *
	**Edge Deformation**	moderate-severe deformation	mild deformation	n/a	moderate-severe deformation	mild deformation	severe deformation	n/a
	**Dilator Removal**	0.437±0.013	0.060±0.020	1.30x10^-15^ *	0.136±0.005	0.043±0.020	1.008±0.305	4.27x10^-6^ *
**Positioning**	**Tip Bending**	3.745±0.380	4.381±0.779	0.076	3.693±0.243	4.165±0.664	1.91±0.452	3.84x10^-8^
	**Buckling**	0.889±0.022	1.031±0.039	1.035x10^-7^ *	1.058±0.023	1.102±0.079	0.271±0.030	4.69x10^-25^
	**Kinking**	4.389	4.137	0.867	3.628	3.767	1.626	0.086
**Lubricity**	**Insertion (biological model)**	—	—		0.467	0.277	0.344	1.42x10^-5^ *
	**Sheath Extraction, biological model (friction)**	0.079±0.010	0.059±0.009	0.004*	0.090±0.031	0.055±0.009	0.260±0.072	3.53x10^-7^ *

* = p-values have no units

#### Tip Perforation

The Terumo sheaths consistently required significantly lower foil perforation forces (GW, 0.398 lbs for 11/13f, and 0.286 lbs for 12/14f) than the NHD (0.67 for 11/13f, 0.628 lbs for 12/14f, p<0.05). Of the 12/14F sheaths, the PW required the least perforation force (p<0.05), and required approximately 1/3 of the perforation force of the NHD (0.245 lbs s 0.628 lbs, p<0.05), but was not significantly different than the GW (0.286, p=0.270).

#### Dilator Removal

The PW sheath had the largest force for dilator removal (1.008 lbs), requiring >20 times more force than the NHD (0.043 lbs, p<0.05) and 7 times more force than GW (0.136, p<0.05). The GW sheath also required significantly greater force for dilator extraction than the NHD. This was consistent across both sheath sizes, with the GW 11/13F requiring 7 times more force (0.437 lbs) than the NHD 11/13F (0.060 lbs, p<0.05), and the GW 12/14F. Frequiring over 3 times more force (0.136 lbs) as the NHD 12/14F (0.043 lbs, p<0.05).

#### Edge Deformation

**Figure 6:**
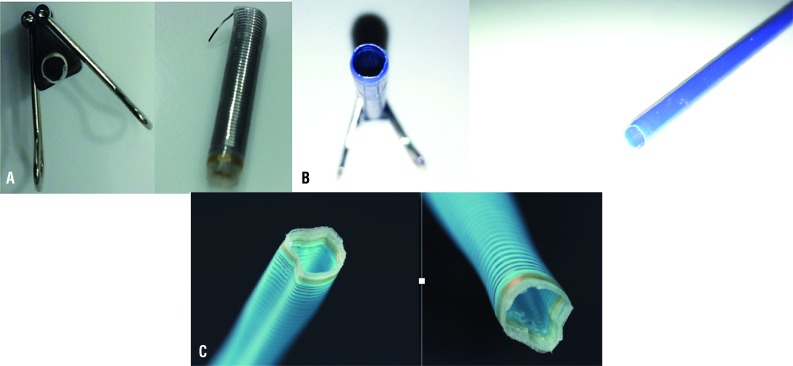
A) Glideway edge deformation; B) Navigator HD edge deformation; C) Pathway edge deformation.

The PW exhibited severe deformation of its edge at a set force of 2.5 lbs. The GW exhibited moderate-severe deformation of its edge at a set force of 2.5 lbs, while the NHD showed only mild deformation at the same force. This occurred at both sheath sizes. Images of deformed sheath edges are displayed in Figures 6A-C.

### Extraction Force for Pathway Sheath (Ex Vivo Model)

**Figure 7:**
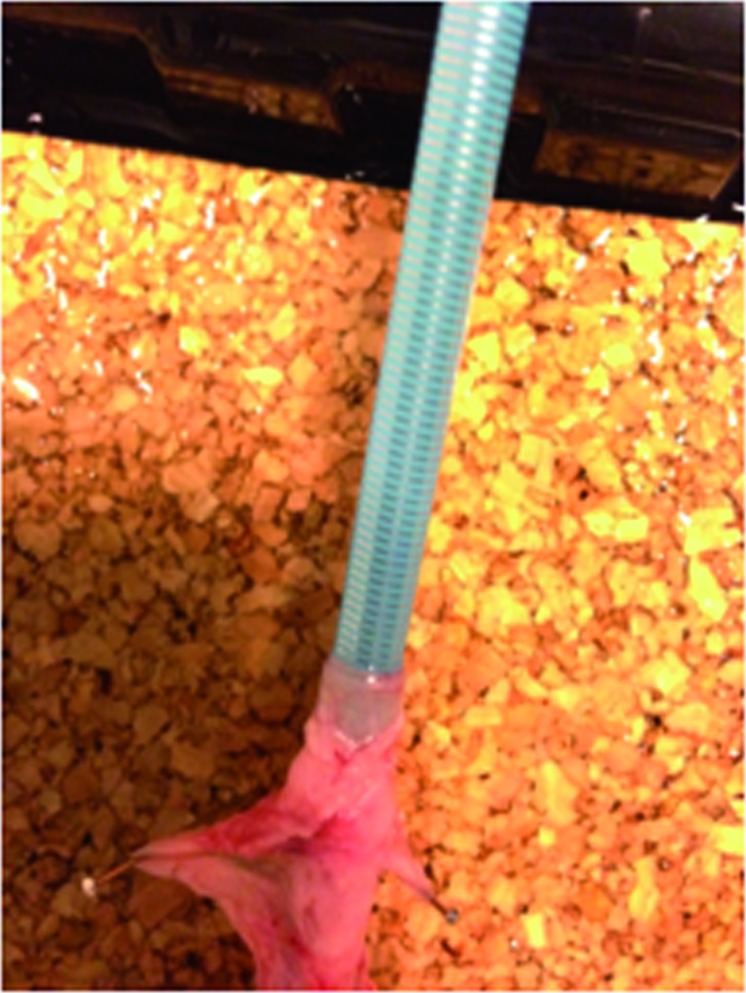
Pig Ureteral Avulsion with Extraction of Pathway.

After the PW was left in place for 30 min. in the pig ureter, it required 0.010 lbs for extraction. After 60 minutes, PW required 0.156 lbs for extraction from the ex vivo model. At both time points, apparent mucosal avulsion occurred for this sheath (Figure-7).

### Positioning- Table 3

#### Tip Bending/Flexibility

The PW 12F had the weakest inner dilator tip, requiring approximately half the force (1.91 lbs) of the other two sheaths (GW 3.69 lbs, NHD 4.17, p<0.05). The GW and NHD inner dilator tips were equally stiff, requiring similar forces for the tip flexibility experiments at both sizes, with the GW 11/13F and 12/14F requiring 3.745 and 3.693 lbs, respectively and NHD 11/13F and 12/14F requiring 4.381 and 4.165 lbs, respectively (p=0.153).

#### Shaft Buckling

The PW sheath was most susceptible to shaft buckling/bending forces, requiring 1/4 of the force (0.271 lbs) of the other sheaths for this experiment (GW 12 1.058, NHD 12 1.202, p<0.05). The two Terumo sheaths required significantly different forces when compared as a pair (p<0.05). The GW 12/14F and NHD 12/14F shafts were equally resistant to coaxial forces, requiring similar forces for sheath buckling (GW 1.058 lbs, NHD 1.102 lbs, p=0.11). However, for the 11/13F sheaths, the NHD was significantly stronger against coaxial forces, requiring 1.031 lbs for sheath buckling, while the GW required 0.889 lbs (p<0.05).

#### Kinking

The PW required the least amount of force for kinking (1.626 lbs), and this was significantly less force than the NHD (3.767 lbs, p<0.05), but not significantly less than the GW (3.628 lbs, p=0.118). Both GW and NHD sheaths were equally resistant to kinking (perpendicular forces), with the GW 11/13F and 12/14F sheaths withstanding 4.389 lbs and 3.628 lbs, respectively and the NHD, 4.137 and 3.767 lbs, (p=0.867 for 11/13f and p=0.92 for 12/14f).

### Insertion Force Through Biological Model

The three sheaths required significantly different mean forces for insertion through the biological model (ANOVA p=1.42x10^-5^). The GW 12 required significantly greater force (0.468 lbs) than both the PW 12 (0.344 lbs, p=0.002) and the NHD 12 (0.277 lbs, 4.29x10-5). The NHD required significantly less force compared to the PW (p=0.02).

### Lubricity- Table 3

The lubricity testing revealed that the NHD sheath consistently required significantly lower frictional forces in a biological model than the GW (0.059 vs. 0.079 lbs, p<0.05 for 11/13F sheaths and 0.055 and 0.090, p<0.05 for 12/14F sheaths) and the PW (0.230 lbs vs. 0.055 for 12f, p<0.05). In fact, the PW required 4 times more frictional force than the NHD.

## DISCUSSION

Though the use of ureteral access sheaths remains a debated topic in endourology, there are many aspects of flexible ureteroscopy that may be facilitated with their use. Several studies have shown that irrigation pressures conducted to the renal pelvis and throughout the kidney are lower during ureteroscopy with a UAS than without a UAS (5, 6, 11). Although the effects of this have not been fully outlined, reduced pressures may have the capacity to prevent the dissemination of infection during treatment of struvite stones as well as in cases of stones with secondary infection (5). The UAS also allows sustained access, permitting easier insertion and extraction of various instruments, and can improve visualization of the procedure (1, 8, 9). It has been shown that UAS use may positively affect outcomes following flexible ureteroscopy (1). In one retrospective study on 256 patients, the UAS resulted in improved stone free rates on IVU or CT at two months following stone procedure (8). In addition, in several other studies, UAS use has been shown to reduce operative time, cost and morbidity (1, 8, 12). Anuway, the studies supporting the use of UAS have low level (3b) of evidence. UAS are available in a variety of sizes and designs and have evolved to fit the ureteroscope. Most UAS have a hydrophilic coating, radio-opaque markings, and a tapered tip with a smooth transition between dilator and outer sheath.

While ureteroscopy is considered a valuable tool in the management of nephrolithiasis, it can result in ureteral injury (13). In a large, single-center study, the overall intra-operative complication rate of ureteroscopy was 3.7%, false passage rates were 1%, and morbidity from ongoing hematuria and renal colic was 2.04% and 2.23%, respectively (13). While the UAS has the safety advantages of improved visualization, reduced renal pressures, and simplified access, the use of a ureteral access sheath does not come without inherent risk of harm. Safety concerns include the risk of ureteral perforation or avulsion, and it has been proposed the UAS use may increase surgical costs, as UAS use mandates use of extra equipment (wires and ureteral stents). In a prospective study of 359 patients undergoing URS, Traxer and Thomas systematically graded and assessed ureteral wall injury resulting from UAS insertion, and found that up to 46.5% of patients had some type of ureteral wall injury (14). While 13.3% of these were “severe” (involving the ureteral smooth muscle), the clinical relevance of the minor injuries is not fully understood (13). As such, it is critical that new sheaths be evaluated systematically for physical characteristics that may impact clinical performance, risk, and outcomes. Previous *in vitro* studies have identified clinically relevant mechanical properties pertaining to UAS usability, such as resistance to buckling and kinking forces, and lubricity (11, 15). Here, we apply these principles to a new set of ureteral access sheaths in order to assess safety characteristics and clinical differences among new equipment.

Although most sheaths have a basic, stiff inner dilator with a flexible tip, Terumo PW has a distinct inner balloon-inflatable model, designed with a smaller distal sheath diameter (deflated balloon) for insertion. Once advanced up the ureter, the balloon is then inflated, and the inner dilator is then removed as with other sheath designs. The intent of this design was to decrease insertion forces and the risk of perforation with a smaller caliber sheath at time of insertion. However, our study demonstrates that insertion forces are similar to traditional sheaths and perforation forces are lower (i.e. the risk of perforation is higher with less force). As such, the PW design does not appear to provide a safety advantage. Instead, this study demonstrates a safety hazard – with a larger extraction force leading to a higher risk of mucosal avulsion.

The Terumo Sheaths (PW and GW) consistently required less force for tip perforation, for both sizes of sheaths. This may be explained by the observation that the inner dilator tips of both the GW and PW were indeed sharper (shallower grade). As such, the GW and PW tips carry a risk of ureteral perforation at lower insertion forces.

The PW required significantly greater force than both the GW and the NHD for inner dilator removal. Mechanical difficulty with dilator extraction could introduce unnecessary tip displacement within the ureter. Although the forces may be small, these unduly large differences in forces (PW >20 times the force of the NHD) implies key differences in usability and design with regard to this step of the procedure. The clinical impact of these differences is unknown. Here, the NHD has the advantage in that its inner dilator can be removed more smoothly.

Safety parameters regarding the PW were further outlined in our *ex vivo* experiment, where outer sheath extraction was evaluated following placement for 30-60 minutes in pig ureters, revealing that gross mucosal avulsion occurs with sheath extraction. Since the extraction forces increased from 30 to 60 min., it is possible that the propensity for ureteral injury increases with duration of surgery.

As displayed in Figures 6A-C, the Terumo sheaths suffered the greatest mechanical damage when impacted against the pseudo calculus, with the PW showing severe deformation and the GW showing moderate to severe deformation, while the NHD only had mild-moderate damage. This experiment simulates an intra-operative attempt to basket a stone that is too large to pass easily through the ureteral access sheath. A sheath that does not uniformly tolerate forces at its edges is more prone to deformation in this setting. The greater edge destruction may correspond to a structural difference in sheath edge, such that a force against the edge does not disperse uniformly. Indeed, we found that the GW's sheath edges were slightly thinner than the NHD's. In theory, the force applied by an impacted stone at the end of the sheath can distort the sheath edges. It is possible that the significant edge damage rendered by the stone for the Terumo PW and GW versus the NHD could prevent extraction of other stones during the procedure, and if severe could disrupt the urothelium during sheath extraction.

In our experiments, the NHD 11/13F sheath was more resistant to buckling than the GW 11/13F sheath. Clinically, this is important during the initial insertion of the sheath into the ureter (12). We can infer that the small-dia-meter NHD sheath may perform better in the case of a difficult insertion into a tight ureteral orifice and would less likely buckle in the bladder.

Newer, more durable ureteral access sheaths have been designed with metal coiling within the sheath to minimize kinking during insertion (15). Both the GW and NHD sheaths performed equally well in response to kinking forces, withstanding large forces with minimal damage. Resis-tance to kinking helps minimize the risk of extrinsic compression at the bladder neck or point of ureteral narrowing (12). However, the PW sheath was significantly more susceptible to kinking forces than the NHD. Inherently, the PW requires a malleable outer sheath design to allow expansion with balloon inflation; as such, the resistance to kinking is lower when compared to the NHD, which possesses a stainless steel coil reinforcement for added strength. A limitation of our study is that not all commercially available ureteral access sheaths were selected for comparison.

The insertion force experiment was designed to compare both ease of sheath placement and propensity for ureteral injury during sheath advancement. The NHD had the lowest maximum insertion force while traversing our biological model, while the GW required the largest insertion force; the PW's force was intermediate. It is possible that the NHD's coating and smoother transition between dilator transition between inner dilator and outer sheath provide for lower insertion forces. Indeed, the NHD is the only sheath available commercially that had a mild taper of the outer sheath's leading edge, likely minimizing the excess force introduced by sudden changes in sheath caliber. The GW's large insertion forces may result from difference in outer coating, or alterations in dilator-sheath transition; as this sheath did not have an outer sheath taper according to our measurements. This quality predisposes the GW to minor mucosal injuries during sheath insertion. The PW's small sheath profile and balloon-inflation mechanism is designed to depend on radial, or circumferential dilation of the ureter in lieu of introducing axial shearing forces, thereby reducing insertion forces, in theory. Here, we demonstrate again that this design does not minimize insertion forces as it claims to, as they are still considerably higher than the NHD. This difference is likely due to the stiffer tip and shaft of the PW.

The friction experiment was designed to assess ease of advancement and smooth handling of the UAS. We hypothesized that the ease of insertion would also correspond to the slipperiness of the outer sheath, as assessed in the friction testing. Indeed, the NHD also required the lowest frictional forces. NHD required significantly less frictional force at both sheath sizes than the GW, and the NHD 12F required 1/4 of the frictional force of the PW 12F. The slippery quality of the NHD sheath may provide for easier, smoother advancement compared to the other sheaths. Since the frictional forces were quite small, the clinical impact of differences in lubricity is unclear.

The differences in radio-opacity among the sheaths may additionally affect ease of sheath placement. While the outer sheath markings of the Terumo sheaths may aid in guiding placement, we are concerned about the poor visibility of the inner dilator tip and the outer sheaths' distal leading edge for both the GW and PW. A poorly visualized dilator tip may lead to unintentional mucosal, renal pelvic or parenchymal injury during placement. The uniformly radiopaque quality of the NHD may aid in placement, ensuring both outer sheath and inner dilator are easily visualized.

## CONCLUSIONS

Comparison of different commercially available UAS in various sizes reveals that there are mechanical differences in sheaths that may play a role clinically. The Terumo sheaths' (GW and PW) were outperformed by the Boston Scientific NHD in simulating safety, ease of use and radio-opacity. The PW's balloon design and smaller distal sheath caliber has no benefit in terms of sheath insertion forces and may pose a risk of mucosal avulsion.

## COMPLIANCE WITH ETHICAL STANDARDS

### Ethical approval

All applicable international, national, and/or institutional guidelines for the care and use of animals were followed.
